# Casein kinase 2 phosphorylation of Hsp90 threonine 22 modulates chaperone function and drug sensitivity

**DOI:** 10.18632/oncotarget.272

**Published:** 2011-05-14

**Authors:** Mehdi Mollapour, Shinji Tsutsumi, Yeong Sang Kim, Jane Trepel, Len Neckers

**Affiliations:** ^1^ Urologic Oncology Branch, Center for Cancer Research, National Cancer Institute, Bethesda, MD 20892, USA; ^2^ Medical Oncology Branch, Center for Cancer Research, National Cancer Institute, Bethesda, MD 20892, USA

**Keywords:** Heat Shock Protein 90, phosphorylation, casein kinase 2, Hsp90 inhibitors, heat shock response

## Abstract

The molecular chaperone Heat Shock Protein 90 (Hsp90) is essential for the function of various oncoproteins that are vital components of multiple signaling networks regulating cancer cell proliferation, survival, and metastasis. Hsp90 chaperone function is coupled to its ATPase activity, which can be inhibited by natural products such as the ansamycin geldanamycin (GA) and the resorcinol radicicol (RD). These compounds have served as templates for development of numerous natural product Hsp90 inhibitors. More recently, second generation, fully synthetic Hsp90 inhibitors, based on a variety of chemical scaffolds, have also been synthesized. Together, 18 natural product and synthetic Hsp90 inhibitors have entered clinical trial in cancer patients. To successfully develop Hsp90 inhibitors for oncology indications it is important to understand the factors that influence the susceptibility of Hsp90 to these drugs *in vivo*. We recently reported that Casein Kinase 2 phosphorylates a conserved threonine residue (T22) in helix-1 of the yeast Hsp90 N-domain both *in vitro* and *in vivo*. Phosphorylation of this residue reduces ATPase activity and affects Hsp90 chaperone function. Here, we present additional data demonstrating that ATP binding but not N-domain dimerization is a prerequisite for T22 phosphorylation. We also provide evidence that T22 is an important determinant of Hsp90 inhibitor sensitivity in yeast and we show that T22 phosphorylation status contributes to drug sensitivity *in vivo*.

## INTRODUCTION

Molecular chaperones are involved in protein folding and homeostasis [1]. Hsp90 is an essential, evolutionarily-conserved molecular chaperone that is ubiquitously expressed both in normal and cancer cells [2-5]. While Hsp90 has been shown to bind to and prevent the aggregation of a wide range of proteins [6], the list of proteins that require active chaperoning by Hsp90 is more restricted (www.picard.ch/downloads/Hsp90interactors.pdf), and is comprised primarily of key components of numerous signal transduction pathways. In cancer cells, Hsp90 plays a vital role in protecting selected mutated, over-expressed and/or deregulated oncoproteins from misfolding and degradation [7, 8]. Therefore it is not surprising that multiple Hsp90 inhibitors are being actively evaluated in the clinic (Table 1) [9].

**Table 1:** Current Hsp90 inhibitors in clinical trials

Inhibitor	Phase	Route	Indication*	Structure	Imaging	Combination drug(s)	Source
Retaspimycin hydrochloride (IPI-504)	II	i.v.	Advanced dedifferentiated liposarcoma, NSCLC patients with ALK translocations	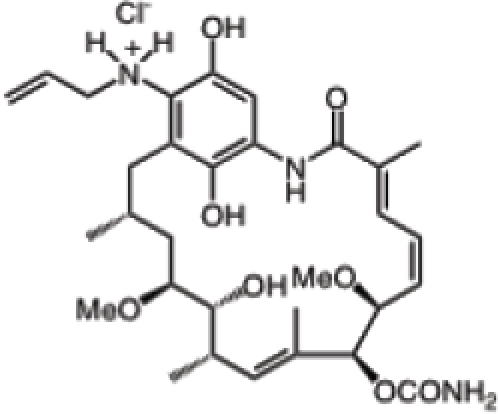	-	-	Infinity
IPI-493	I	oral	Advanced solid tumors, hematologic malignancies	-	-	-	Infinity
AUY922	I/II	i.v.	Advanced solid malignancies, MBC, stage IIIB-IV NSCLC, advanced gastric cancer, metastatic colorectal cancer	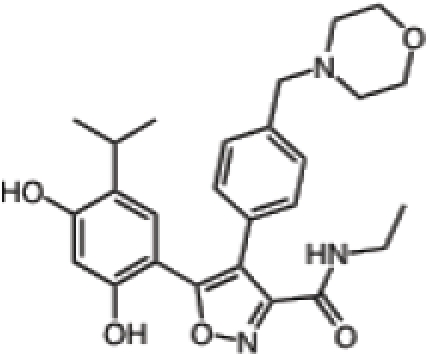	^89^Zr-trastuzumab, VEGF-^89^ZR-bevacizumab PET imaging	Trastuzumab, erlotinib, capecitabine, cetuximab	Novartis
Ganetespib (STA-9090)	I/II	i.v.	AML, MDS, CML, or myeloproliferative disorders, stage IIIB or IV NSCLC, GIST, colorectal cancer, SCLC, esophagogastric cancer, metastatic ocular melanoma, metastatic pancreatic cancer, metastatic HRPC, MBC, solid tumors	-	-	Docetaxel	Synta
KW-2478	I/II	i.v.	MM	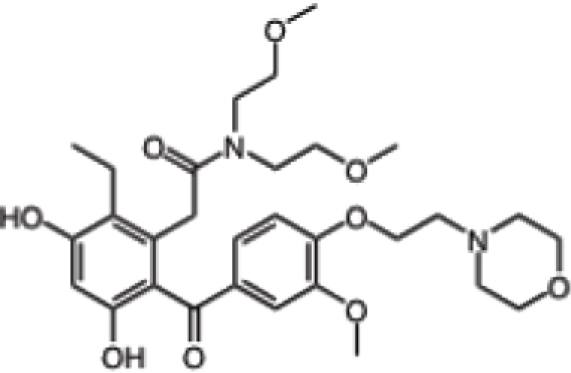	-	Bortezomib	Kyowa Hakko Kirin
AT13387	I/II	oral i.v.	GIST, metastatic solid tumors	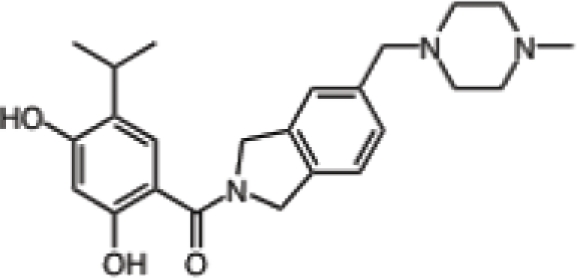	-	Imatinib	Astex Therapeutics
HSP990	I	oral	advanced solid tumors	-	-	-	Novartis
MPC-3100	I	oral	Cancer patients who have failed other treatments	-	-	-	Myrexis, Inc.
DS-2248	I	oral	Advanced solid tumors	-	-	-	Daiichi Sankyo Inc.
Debio 0932	I	oral	Advanced solid tumors or lymphoma	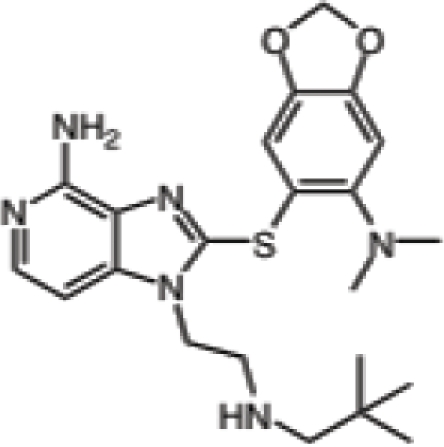	-	-	Debiopharm/Curis
PU-H71	-	i.v.	Breast cancer, prostate cancer or lymphoma	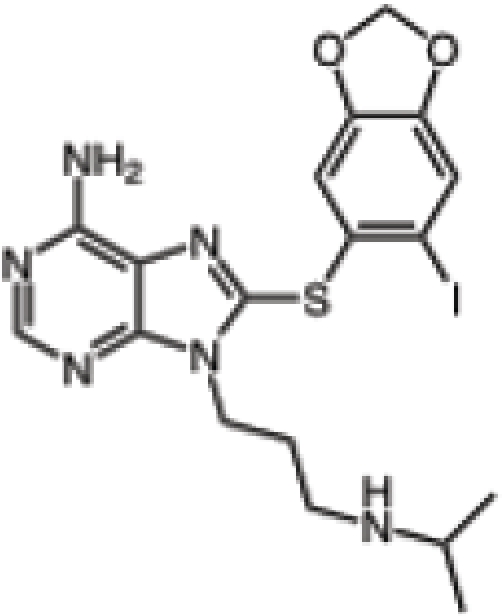	[^124^I]-PU-H71 PET imaging	-	Memorial Sloan-Kettering Cancer Center
* non-small cell lung cancer (NSCLC), small cell lung cancer (SCLC), chronic lymphocytic leukemia (CLL), small lymphocytic lymphoma (SLL), or B-cell prolymphocytic leukemia (B-PLL), acute myelogenous leukemia (AML), myelodysplastic syndrome (MDS), chronic myelogenous leukemia (CML), gastrointestinal stromal tumor (GIST), metastatic breast cancer (MBC), multiple myeloma (MM), hormone-resistant prostate cancer (HRPC).							

**Figure 1:**
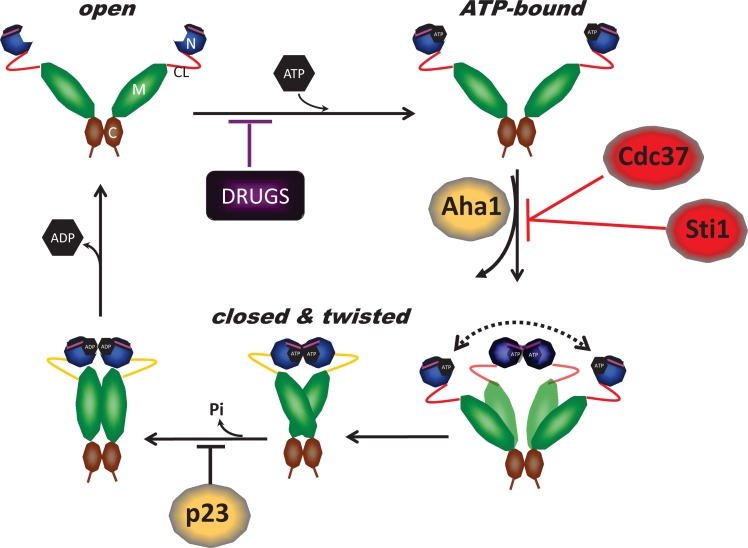
The Hsp90 chaperone cycle Hsp90 is a dimer of two monomers, each containing an N-domain (“N”, blue); a charged linker (“CL”, red); an M-domain (“M”, green); and a C-domain (“C”, brown). The ATP lid (purple) in the N-domain alters conformation upon ATP binding to the N-terminal domains of the “open” conformation, promoting “lid” closure followed by transient dimerization of the N-domains. Subsequent structural changes result in the “closed and twisted” conformation that is competent for ATP hydrolysis. The co-chaperones Sti1 and Cdc37, and pharmacologic inhibitors such as those discussed in the text, slow or block, respectively, the chaperone cycle at an early stage, while the co-chaperone Aha1 enhances the ATPase activity of Hsp90 by assisting the extensive and energy-intensive conformational changes necessary to achieve competence for ATP hydrolysis. The co-chaperone p23 stabilizes a late Hsp90 conformation and slows, but does not block, the rate of ATP hydrolysis. For more details, please see [4].

Hsp90 is dimeric and each protomer can be divided into three domains (Figure 1) [10, 11]: i) an N-terminal domain, containing nucleotide, co-chaperone (proteins that assist the chaperone activity of Hsp90), and drug binding sites; ii) a middle (M) domain, which provides binding sites for client proteins and co-chaperones; iii) a C-terminal domain containing a dimerization motif, a second inhibitor binding region and binding sites for additional co-chaperones [12-14]. N and M domains are connected by an unstructured charged-linker region of significant but variable length, which provides conformational flexibility to the protein [15, 16].

Hsp90 chaperone function is coupled to its ability to bind and hydrolyze ATP, which in turn promotes an ordered series of conformational changes known as the chaperone cycle that is necessary for Hsp90's chaperone function [17, 18]. Hsp90 inhibitors currently in clinical evaluation all share the property of preventing the chaperone cycle by occupying Hsp90's N-domain ATP binding pocket [19, 20]. The regulation of eukaryotic Hsp90 function is complex and depends on several factors, including the regulated interaction of specific co-chaperones (e.g., Aha1 stimulates Hsp90 ATPase activity, whereas p23/Sba1 and Hop/Sti1 inhibit ATP hydrolysis [21, 22]), and various post-translational modifications [23, 24]. A more detailed understanding of the mechanics of Hsp90 regulation in normal and cancer cells may provide additional therapeutic strategies to effectively inhibit this protein.

## HSP90 PHOSPHORYLATION REGULATES CHAPERONE FUNCTION

Hsp90 is subject to several post-translational modifications, including phosphorylation, acetylation, and S-nitrosylation, that contribute to Hsp90 regulation, although regulation of these processes within the cell is not well understood [3, 4]. While Hsp90 acetylation and S-nitrosylation have been identified more recently [23, 24], Hsp90 phosphorylation was first reported in the early 1980s [25].

Hsp90 is a substrate for several serine/threonine and tyrosine kinases, including double-stranded DNA-dependent protein kinase, Akt, B-Raf, Protein Kinase A (PKA), Casein Kinase 2 (CK2), c-Src and Wee1 [3, 4, 26] (Table 2). These kinases are also clients of Hsp90, suggesting the possible existence of complex feedback loops whereby these kinases may modulate their own chaperoning and functional activity. Indeed, others have suggested that client binding to Hsp90 may directly influence its chaperone activity [2, 27]. Previous work has shown that brief exposure of cells to okadaic acid, a serine/threonine phosphatase inhibitor, leads to Hsp90 threonine hyperphosphorylation and is accompanied by decreased Hsp90 association with its client kinase v-Src [28]. These data suggest that Hsp90 threonine phosphorylation is tightly regulated and therefore is likely to impact chaperone function.

**Table 2:** Hsp90 phosphorylation sites and identified kinases Yeast (Hsc82, Hsp82) and human (Hsp90α, Hsp90β) phosphorylation residues are marked by an asterisk (*); orthologue residue is also shown when present. These phosphorylation sites were taken from published literature and the websites PhosphoSitePlus® (http://www.phosphosite.org/) and *Saccharomyces* Genome Database (SGD) (http://www.yeastgenome.org/). Marked phosphorylation residues (†) were identified in rat and (§) mouse Hsp90

Residues					
yHsp90	yHsc90	hHsp90a	hHsp90b	Protein Kinase	Reference
N/A	N/A	T5*	N/A	Ds-DNA activated protein kinase	[43]
N/A	N/A	T7*	N/A	Ds-DNA activated protein kinase	[43]
T22*	T22	Y36	Y31	Casein kinase II	[33]
Y24*	Y24	Y38*	Y33	Swe1/Wee1	[29]
S36	S36	S50	S45*	UNKNOWN	[44, 45]
N/A	N/A	S52*	N/A	UNKNOWN	[46]
S39	S39	S53	S48*	UNKNOWN	[44, 45]
Y47	Y47	Y61	Y56*	UNKNOWN	[47-50]
S49	N/A	S63*§	S58*	Casein kinase II	[49, 51]
S51	S51	T65*	T60	Casein kinase II	[51]
N/A	N/A	S68*	S63	Casein kinase II	[51]
T58	T58	S72*	S67	Casein kinase II	[51]
N/A	N/A	T88*	T83	PKA	[52]
N/A	N/A	T90*	T85	UNKNOWN	[53]
Y184	Y184	Y197	Y192*	UNKNOWN	[48]
S198	S198	S211†	S206	PKA, PKG	[54]
N/A	N/A	S231*	S226*	Casein kinase II	[31, 55, 56]
N/A	N/A	S252*	N/A	UNKNOWN	[57]
N/A	N/A	S263*	S255*	Casein kinase II, B-Raf	[31, 55, 56, 58]
N/A	N/A	N/A	S261*†§	UNKNOWN	[49, 57, 59-64]
N/A	N/A	Y284*	Y276*	UNKNOWN	[50, 65]
S282*	S278	N/A	N/A	UNKNOWN	SGD
T273	T269	T293*	T285*	UNKNOWN	[66]
T285	T281	T305	T297*	UNKNOWN	[67]
S297*	S293	N/A	N/A	UNKNOWN	SGD
Y289	Y285	Y309	Y301*	c-Src (does not exist in yeast)	[68]
Y293	Y289	Y313§	Y305*	UNKNOWN	[69]
S295	S291	S315	S307*	UNKNOWN	[63]
S297	S293	T317*	T309*	UNKNOWN	[66]
S334*	S330	N/A	N/A	UNKNOWN	SGD
S379*	S375	S399	S391*	UNKNOWN	SGD, [49, 59, 65]
T443*	T429	S453§	S445*	UNKNOWN	SGD, [59]
N/A	N/A	N/A	T446§	UNKNOWN	[49, 59]
N/A	N/A	S460	S452*	PKA	[54]
N/A	N/A	Y466*	N/A	UNKNOWN	[50], PhosphoSitePlus®
S456	S452	S476§	S468	UNKNOWN	[49]
N/A	N/A	N/A	S482§	UNKNOWN	[46]
Y472	Y468	Y492*	Y484*	UNKNOWN	[45, 70], PhosphoSitePlus®
Y473	Y469	Y493*	Y485*	UNKNOWN	PhosphoSitePlus®
S478	S474	T495§	S490	UNKNOWN	[45]
T502	T498	N/A	T514§	UNKNOWN	[45]
Y508	Y504	Y498	Y520§	UNKNOWN	[45]
T520	T516	T540	S532*	UNKNOWN	[63]
N/A	N/A	T566§	N/A	UNKNOWN	[49]
S568	S564	S589*	S581	UNKNOWN	[66]
N/A	N/A	Y604*§	Y596*	UNKNOWN	[71] PhosphoSitePlus®
Y606	Y602	Y627*§	Y619*	UNKNOWN	PhosphoSitePlus®
S616*	S612	N/A	N/A	UNKNOWN	SGD
S619*	S615	N/A	N/A	UNKNOWN	SGD
N/A	N/A	S641§	N/A	UNKNOWN	[49]
S657*	S653	S677	S668	UNKNOWN	[72]
S663*	S659	N/A	N/A	UNKNOWN	[72]
N/A	N/A	Y689*	Y681	UNKNOWN	[50], PhosphoSitePlus®
N/A	N/A	T725*	N/A	UNKNOWN	[63]
N/A	N/A	S726*	S718*	UNKNOWN	[63]

We reported recently that Wee1^Swe1^ phosphorylates a conserved tyrosine residue (Y24 in yeast Hsp90 and Y38 in human Hsp90α) in the N-domain of Hsp90 in a cell cycle-dependent manner [29]. Interestingly, deletion of Swe1 in yeast or pharmacologic inhibition of Wee1 in cancer cells confers hypersensitivity to Hsp90 inhibition [29].

## CK2 DEPENDENT PHOSPHORYLATION OF HSP90

CK2 is a serine/threonine protein kinase and an Hsp90 client [30] that phosphorylates multiple serine and threonine residues in human Hsp90α (hHsp90α) and yeast Hsp82 (yHsp90) [31, 32]. Recently, we used *S. cerevisiae* to show that CK2 phosphorylates a single conserved threonine residue (T22) in the N-domain of yHsp90 both *in vitro* and *in vivo* [33]. T22 resides in helix-1 of the Hsp90 N-domain that, together with other adjacent amino acids, is involved in an important hydrophobic interaction with the ATPase catalytic loop in the M-domain that helps to establish Hsp90's ATP hydrolysis-competent state.

The functional importance of T22 was uncovered initially in a genetic screen, where its mutation to isoleucine affected the chaperoning of v-Src and glucocorticoid receptor (GR) in yeast [34]. Subsequent work demonstrated that the T22I mutant has 6-fold higher ATPase activity compared to WT yHsp90 [35]. In contrast, we have shown that mutation of T22 to non-phosphorylatable alanine (T22A) did not affect its ATPase activity, while phospho-mimetic mutation of this residue to glutamic acid (T22E) reduced ATPase activity by 60% compared to WT yHsp90. Nevertheless, both mutants in yeast and the equivalent mutations in hHsp90α (T36A and T36E) affected Hsp90-dependent chaperoning of kinase (v-Src, Mpk1/Slt2, Raf-1, ErbB2 and CDK4) and non-kinase (cystic fibrosis transmembrane conductance regulator protein and GR) clients [33].

**Figure 2:**
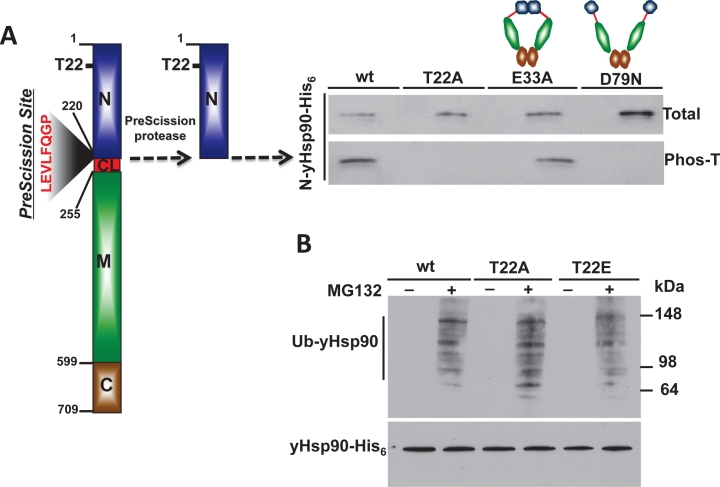
A) CK2-mediated threonine phosphorylation of the N-domain of WT, T22A, E33A and D79N yHsp90-His_6_ *in vitro*. Threonine phosphorylation was detected with a pan anti-phosphothreonine antibody. For more details, please see [33]. B) WT, T22A, and T22E yHsp90-His_6_-expressing yeast were treated with the proteasome inhibitor MG132 (50 mM for 1 h), and yHsp90 ubiquitination was assessed using anti-ubiquitin antibody to probe salt-stripped (0.5 M NaCl) His_6_ pull-downs.

To explore further whether ATP binding is a prerequisite for T22 phosphorylation, we examined the ability of CK2 to phosphorylate two conformationally distinct Hsp90 N-domain mutants. CK2 was able to efficiently phosphorylate yHsp90-E33A, which binds ATP equivalently to wild-type but, upon ATP binding, favors a “closed” (N-domain dimerized) conformation (i.e., is unable to hydrolyze ATP). However, CK2 was unable to phosphorylate yHsp90-D79N, which cannot bind ATP and thus favors an “open” (N-domain undimerized) conformation (Figure 2A). Since T22 is not accessible to solvent once ATP-dependent N-domain dimerization has occurred [18], these data suggest that ATP binding to the open conformation of Hsp90 sets in motion rapid conformational change within and adjacent to helix-1 that is a prerequisite for CK2-mediated phosphorylation. Indeed, a recent study of the bacterial ortholog of Hsp90, HtpG, confirms that this region of the N-domain undergoes very rapid conformational change upon ATP binding that significantly precedes ATP-induced N-domain dimerization [35, 36] Since T22 phosphorylation slows the rate of ATP hydrolysis without affecting ATP binding, it is possible that eukaryotic cells utilize this post-translational modification to adjust the rate of the Hsp90 cycle to meet the optimal chaperone requirements of individual client proteins.

We showed previously that phosphorylation of Y24 is a signal for Hsp90 polyubiquitination and degradation by cytoplasmic proteasomes [33]. We explored the possibility that T22 phosphorylation of yHsp90 may serve a similar purpose. Yeast expressing WT Hsp90 as well as the phospho mutants (T22A and T22E) were treated with the proteasome inhibitor MG132 (50 μM for 1h). This resulted in equivalent accumulation of polyubiquitinated yHsp90 in each case (Figure 2B). Therefore, unlike Y24, T22 phosphorylation is not likely to be a determinant of Hsp90 degradation. Instead, one can speculate that T22 phosphorylation occurs dynamically to allow for the fine-tuning of chaperone activity in response to environmental cues.

## CK2 PHOSPHORYLATION OF THREONINE 22 IMPACTS CHAPERONING OF HEAT SHOCK FACTOR (HSF)

**Figure 3:**
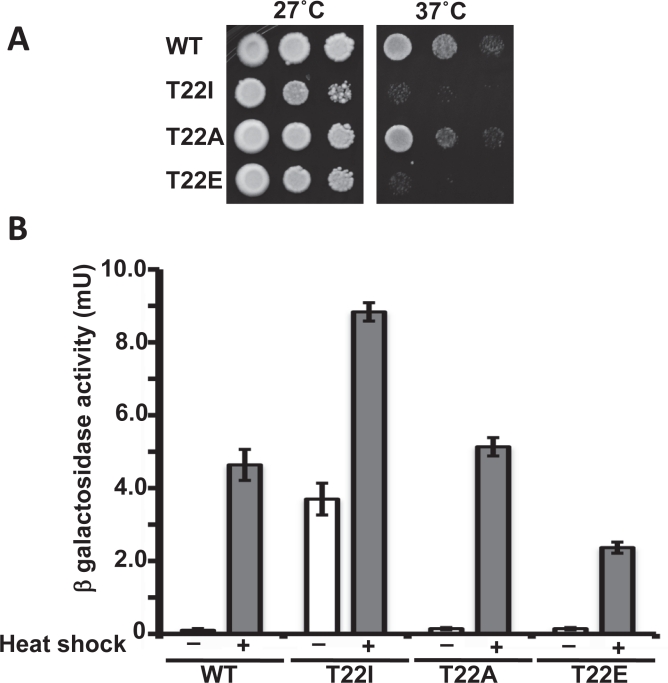
A) Yeast expressing WT yHsp90-His_6_ or the phospho mutants T22A and T22E, as well as T22I, were grown to mid-log phase (10^7^ cells/ml) and then a 1:10 dilution series was spotted on YPDA agar. The plates were incubated either at 27°C or 37°C for 3 days to test for temperature sensitivity. B) Yeast cells expressing WT yHsp90-His_6_ or phospho mutants T22A and T22E, or T22I, were transformed with a Heat Shock Element (HSE)-*lacZ* reporter. HSE activity was measured in unstressed (light bars) and heat shocked (40 min at 39°C, dark bars) cells. The data are expressed as mean +/− standard deviation derived from four independent experiments.

The phospho-mimetic T22E mutant, like T22I, is temperature sensitive whereas T22A-expressing yeast grow like wild-type cells at elevated temperature (Figure 3A). This phenotype may reflect the different ATPase activities of these mutants, since it is generally accepted that Hsp90 binding serves to down-regulate *HSF* transcriptional activity [37]. Mutations of Hsp90 that compromise its chaperone function, or Hsp90 inhibitor administration, lead to strong induction of Hsf1 activity in yeast, even in the absence of heat shock [38].

To examine the importance of T22 in Hsp90 modulation of the heat shock response, we measured Hsf1 activity in yHsp90 T22A (normal ATPase activity), T22E (reduced ATPase activity) and T22I (elevated ATPase activity)-expressing yeast cells by transforming them with a heat shock element HSE-*lacZ* reporter plasmid. We confirmed that yHsp90-T22I displayed a higher basal and heat-induced (39°C for 40 min) Hsf1 activity compared to WT cells [39] (Figure 3B), while Hsf1 activity was significantly diminished in the phospho-mimetic yHsp90-T22E mutant. At the same time, Hsf1 activity in yHsp90-T22A mutant yeast was equivalent to that of yeast expressing WT yHsp90. These data suggest that the ability of yHsp90 to regulate Hsf1 closely parallels its inherent ATPase activity, but that temperature sensitivity itself is not an accurate predictor of the impact of these Hsp90 mutations on the heat shock response.

## PHOSPHORYLATION OF THREONINE 22 CONFERS INCREASED SENSITIVITY TO HSP90 INHIBITORS

There are currently 18 Hsp90 inhibitors in various stages of clinical evaluation as targeted anti-cancer agents. Uncovering modifications to Hsp90 that might enhance sensitivity to these inhibitors at the cellular level would certainly aid their continued clinical development. To that end, we have examined the sensitivity of T22 mutants to Hsp90 inhibitors. We expressed non-phospho (T22A) and phospho-mimetic (T22E) Hsp90 mutants, as well as yHsp90-T22I, as the sole Hsp90 species in the PP30 yeast strain lacking the pleiotropic drug resistance pump, Pdr5 [40, 41]. Although the *S. cerevisiae* genome contains 30 plasma membrane ATP-binding cassette (ABC) proteins [42], Pdr5 has been identified as the major mediator of Hsp90 inhibitor efflux [40, 41].

**Figure 4:**
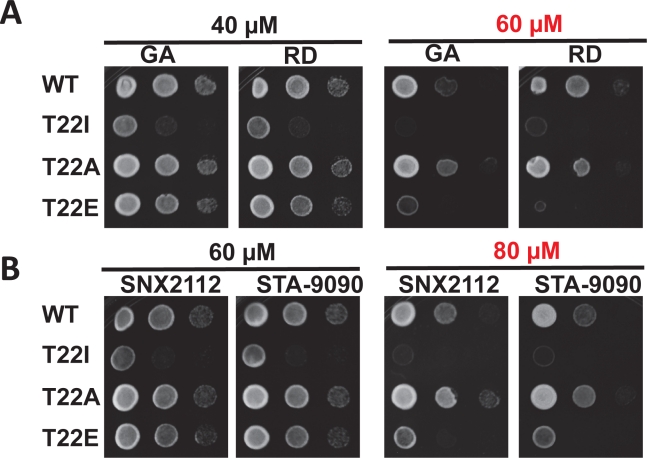
PP30 *pdr5Δ* yeast strain expressing only WT y Hsp90, yHsp90-T22I, yHsp90-T22A, or yHsp90-T22E were grown to mid-log phase (107 cells/ml) and then a 1:10 dilution series was spotted on YPDA agar containing indicated concentrations of the Hsp90 inhibitors geldanamycin (GA), radicicol (RD), SNX2112, or STA-9090 Plates were incubated at 25°C for 4 days.

Yeast cells were grown to exponential phase and were spotted at 10^7^, 10^6^ and 10^5^ cells/ml on YPDA plates containing 40 or 60 μM GA or radicicol (RD). The synthetic Hsp90 inhibitors, ganetespib (formerly STA-9090, Synta Pharmaceuticals) and SNX-2112 (Serenex/Pfizer) were also included in these assays (at 60 or 80 μM). Our data show that the drug sensitivity of y-Hsp90-T22A-expressing yeast was equivalent to or less than that of yeast expressing WT yHsp90. In contrast, yeast expressing yHsp90-T22I were uniformly hypersensitive to all of the Hsp90 inhibitors examined (Figure 4A, B). Interestingly, yeast expressing the phospho-mimetic yHsp90-T22E were also more sensitive than either WT or yHsp90-T22A-expressing yeast to the four Hsp90 inhibitors (Figure 4A, B). However, this increased sensitivity was evident only at higher drug concentrations. These data identify T22 as an important determinant of Hsp90 inhibitor sensitivity in yeast and suggest that T22 phosphorylation status may contribute to drug sensitivity *in vivo*.

## CONCLUDING REMARKS

**Figure 5:**
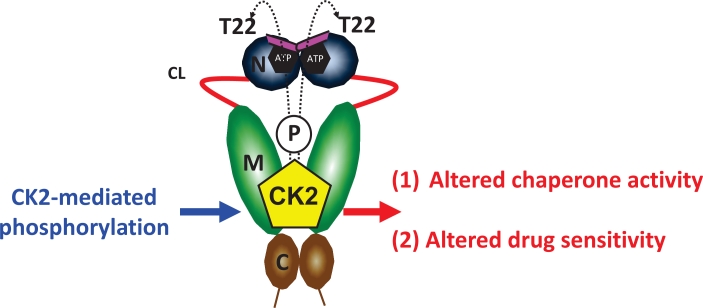
Casein kinase 2 (CK2) phosphorylates a conserved threonine residue (T22) in the N-domain of yHsp90 This residue is an important determinant of Hsp90 ATPase activity and drug sensitivity; T22 phosphorylation affects Hsp90 chaperone function and alters its sensitivity to Hsp90 inhibitors. For further details, please see [33].

Hsp90 phosphorylation has been known for nearly 30 years [25], however only recently are we beginning to appreciate its significance in fine-tuning Hsp90 chaperone activity [4]. Our recent work has demonstrated that the Hsp90 client kinase CK2 phosphorylates a conserved threonine residue (T22) in the N-domain of yHsp90. This residue participates in a hydrophobic interaction with lysine 378 of the catalytic loop in the Hsp90 middle domain, helping to stabilize the ATPase-competent state induced by ATP binding to the N-domain. Phospho-mimetic mutation of this residue in both yeast and human Hsp90 alters chaperone function (see Figure 5 and [33]). Here, we have provided additional data showing that mutation of this residue impacts both the heat shock response and sensitivity to Hsp90 inhibitors.

It is unlikely that the regulation of Hsp90 function, in all its complexity, is universal for all client proteins of this chaperone. Indeed, a recent report [2] has suggested that different clients and co-chaperone complexes can be accommodated by subtly different Hsp90 conformational states. Perhaps phosphorylation of Hsp90, at T22 and additional amino acid residues, participates in modulating the equilibrium among these client-dependent conformational states.
